# Ultrasound-Assisted Deep Eutectic Solvent Extraction of Anthocyanins from Blueberry Wine Residues: Optimization, Identification, and HepG2 Antitumor Activity

**DOI:** 10.3390/molecules25225456

**Published:** 2020-11-20

**Authors:** Hongkun Xue, Jiaqi Tan, Qian Li, Jintian Tang, Xu Cai

**Affiliations:** 1 Key Laboratory of Particle & Radiation Imaging, Ministry of Education, Department of Engineering Physics, Tsinghua University, No. 30 Shuangqing Road, Haidian District, Beijing 100084, China; xuehk0906@163.com (H.X.); liqianb116@163.com (Q.L.); jttanglabb116@163.com (J.T.); 2 Academy for Advanced Interdisciplinary Studies, Peking University, No. 5 Yiheyuan Road, Haidian District, Beijing 100871, China; tanjiaq@pku.edu.cn

**Keywords:** blueberry wine residues, anthocyanins, ultrasound-assisted deep eutectic solvent extraction, optimization, antitumor activity

## Abstract

Blueberry wine residues produced during the wine-brewing process contain abundant anthocyanins and other bioactive compounds. To extract anthocyanins from blueberry wine residues more efficiently, a novel procedure of ultrasound-assisted deep eutectic solvent extraction (UADESE) was proposed in this work. The extraction process was optimized by response surface methodology coupled with genetic algorithm. The optimum extraction parameters to achieve the highest yield of anthocyanins (9.32 ± 0.08 mg/g) from blueberry wine residues by UADESE were obtained at water content of 29%, ultrasonic power of 380 W, extraction temperature of 55 °C, and extraction time of 40 min. The AB-8 macroporous resin combined with Sephadex LH-20 techniques was used to purify the crude extract (CE) obtained under optimum extraction conditions and analyze the anthocyanins composition by HPLC-ESI-MS/MS. The cyanidin-3-rutinoside with purity of 92.81% was obtained. The HepG2 antitumor activity of CE was better than that of the purified anthocyanins component. Moreover, CE could increase the intracellular reactive oxygen species levels and the apoptosis, and arrest HepG2 cells in the S phases. These findings provided an effective and feasible method for anthocyanins extraction, and reduced the environmental burden of this waste.

## 1. Introduction

“Blomidon” blueberry (*Vaccinium* spp.), as a good source of bioactive compounds, includes polyphenols, anthocyanins, superoxide dismutases and flavanols [1]. Blueberries are favored by consumers because of their rich bioactive compounds and strong antioxidant ability [2]. In addition, the consumption of foods enriched in bioactive compounds may be related to reduction in the risk of cardiovascular disease and chronic inflammations, and improvement immunity [3]. Blueberries are suitable for processing into blueberry juice, blueberry wine, and blueberry jam owing to their properties of acidity and sweetness. The skin of blueberry is rich in anthocyanins. Consequently, blueberry wine residues include these bioactive compounds. However, these organic residues, as byproducts and waste materials, are often discarded or processed into cheap animal feed, which leads to increase processing costs. Moreover, the improper handling of residues has a negative effect on the environment. Therefore, the utilization of blueberry wine residues to retrieve bioactive compounds has become a focus of the blueberry processing industry. Finding efficient and environmentally friendly extraction techniques for extracting anthocyanins from natural plant resources has been a challenging task over the last decades.

The anthocyanins extraction from natural plant resources used conventional solvents extraction (ethanol, methanol, *n*-butanol, etc.), which caused excessive solvent consumption and environmental pollution [4]. Deep eutectic solvents (DESs), as a novel extraction solvent, are usually based on mixtures of relatively cheap and easily available components, such as nontoxic hydrogen bond acceptors (HBA) with naturally derived uncharged hydrogen bond donors (HBD), that provide a “green profile” and have broad application prospects in the field of green technologies [5]. Currently, DESs, as a new generation of liquid salts to replace traditional solvents, have attracted extensive attention in various industrial fields, including the extraction of bioactive compounds from various natural plant sources. Moreover, another principle of green extraction is decreased energy consumption by using potential innovative technologies, such as ultrasound. Ultrasound-assistant extraction (UAE) can provide a vital energy source to extract phenolic compounds, and the UAE has many merits of shorter extraction time and lower solvent consumption compared with conventional solvents extraction [6]. In addition, the UAE is commonly performed at lower temperatures, which can avoid the thermal degradation of bioactive compounds caused by high temperature [6]. The combination of UAE and DESs extraction techniques has the merits of both methods, thereby providing effective and environmentally friendly alternatives to the traditional anthocyanins’ extraction method. However, the ultrasonic-assisted deep eutectic solvents extraction (UADESE) is yet to be investigated for blueberry wine residues so for.

The yield of bioactive compounds from different plant materials depends on many factors (extraction temperature, extraction time, solvent properties, etc.) that affect the extraction process. Hence, to achieve the highest yield of anthocyanins from blueberry wine residues, optimizing the extraction process parameters is essential. Response surface methodology (RSM), as a mathematical and statistical method, is widely employed to investigate the individual and the interaction effects of experimental factors on the target compounds yield [7]. Additionally, RSM can effectively reduce the experimental trials, cost and time. However, RSM has a high requirement for the selection of experimental points. If the experimental points are not selected properly, RSM will get unsatisfactory optimization results. The genetic algorithm (GA) has the characteristic of global optimization, which can achieve improved prediction and optimization effects. The combination of RSM and GA can avoid RSM defects and give full play to the advantages of GA in global optimization.

Increasing researches have reported that anthocyanins from raw plant materials were separated and purified using high-performance liquid chromatography (HPLC), column chromatography (CC) and high-speed counter-current chromatography (HSCCC) [8,9,10,11]. However, the separation and the purification of anthocyanins from blueberry wine residues by UADESE has not yet been reported. In terms of anthocyanins activities, the studies are focused on the antioxidation and anti-inflammatory activities of anthocyanins. Nevertheless, the HepG2 antitumor activity of anthocyanins extract and purified anthocyanins components from blueberry wine residues have not yet been reported.

This study aims to: (1) optimize the extraction conditions for the UADESE anthocyanins from blueberry wine residues by using RSM coupled with GA; (2) analyze the anthocyanins composition of the crude extract (CE) obtained under optimum extraction conditions by using HPLC-ESI-MS/MS, and (3) evaluate the HepG2 antitumor activity of the CE and the purified anthocyanins component.

## 2. Results and Discussion

### 2.1. Screening of Deep Eutectic Solvents (DESs) System for the Extraction of Anthocyanins

**Figure 1:**
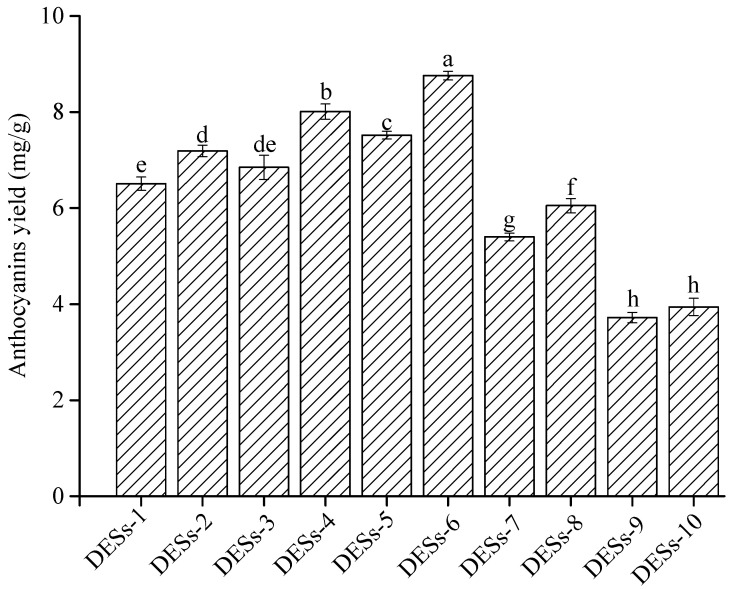
Effect of different deep eutectic solvents (DESs) on the yield of anthocyanins from blueberry wine residues. Note: Different lowercase letters indicate significant differences between groups, *p* < 0.05.

Figure 1 showed the yield of anthocyanins from blueberry wine residues extracted with 10 types of DESs. Results indicated that the best extraction anthocyanins yield followed the order: DESs-6 > DESs-4 > DESs-5 > DESs-2 and DESs-3 > other DESs, which may be because the extraction efficiency depends on the polarity of the anthocyanins. The polarity of DESs-6 was similar to that of anthocyanins. According to the rule “like dissolves like”, anthocyanins were prone to dissolve in DESs-6. In addition, DESs-6 had low viscosity and surface tension, improving the permeability to cells for dissolving anthocyanins. The effects of DESs-6 and DESs-4 on the anthocyanins yield showed that the position of the hydroxyl in the polyol also affected the viscosity and the polarity of DESs, thereby affecting the extraction efficiency. At the same HBD and HBA, DESs with the mole ratio of 1:3 had lower viscosity and surface tension than DESs with the mole ratio of 1:2, which could easily extract the solvent penetration and promote the dissolution of anthocyanins from blueberry wine residues. Therefore, DESs-6 (1,4-butanediol as HBD, the mole ratio of 1:3) was selected as the best extraction solvent to perform the subsequent experiments.

### 2.2. Single-Factor Experiments for Anthocyanins Extraction

**Figure 2:**
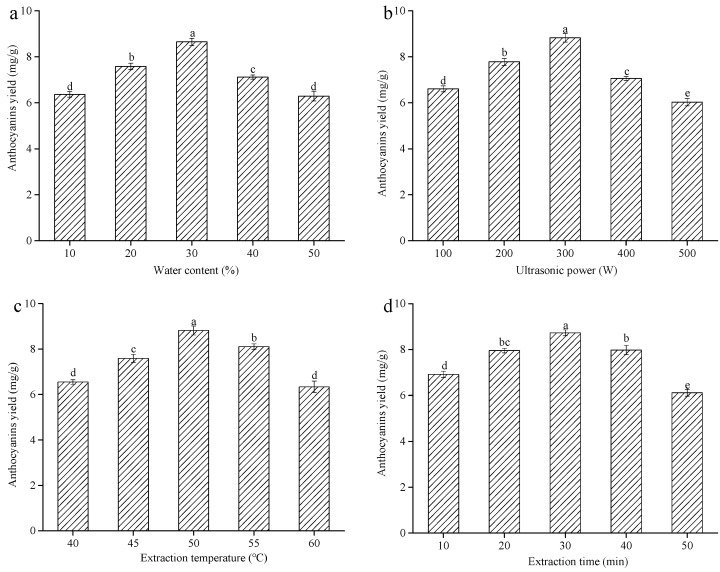
The effects of different extraction variables on the anthocyanins yield from blueberry wine residues using DESs-6 as a eutectic solvent: (**a**) water content, (**b**) ultrasonic power, (**c**) extraction temperature, (**d**) extraction time. Note: different letters indicate significant difference (*p* < 0.05, the same below).

The water content can change the physicochemical properties of DESs, especially viscosity and polarity. Choosing a suitable water content is beneficial for improving the mass transfer rate and the anthocyanins yield. Thus, the effect of different water contents (10%, 20%, 30%, 40%, and 50%) on the yield of anthocyanins from blueberry wine residues was further investigated. It could be seen from Figure 2a that the yield of anthocyanins memorably increased at the water content of 10%–30% (*p* < 0.05). This effect might be because the addition of water effectively weakened the hydrogen bonding of DESs, resulting in a rapid decrease in viscosity [12], which was conducive to improve the solubility and the mass transfer rate of anthocyanins. Nevertheless, the anthocyanins yield significantly decreased when the water content continuously increased from 30% to 50% (*p* < 0.05), which was because the excessively high water content increased the DESs polarity and decreased the solubility of anthocyanins in the DESs [11]. Thus, 20%, 30%, and 40% water contents were used in the subsequent experiments.

Increasing studies have confirmed that ultrasound power could significantly affect the yield of anthocyanins [13]. Hence, experiments were performed to investigate the effect of ultrasound power on the anthocyanins yield. The yield of anthocyanins markedly increased with increasing ultrasound power from 100 to 300 W (*p* < 0.05) and reached the maximum anthocyanins yield (8.83 ± 0.19 mg/g) at 300 W (Figure 2b). This phenomenon occurred because the ultrasonic waves generated a cavitation effect and localized pressure in the solvent, causing faster movement of molecules and improved penetration of the solvent into the blueberry wine residues, which promoted the release of intracellular anthocyanins into the solvents and improved the anthocyanins yield [14]. However, once the ultrasound power was over 300 W, the yield of anthocyanins substantially declined (*p* < 0.05), which was because the high ultrasound power produced excessive heat generation in the reaction systems, This heat generation resulted in the degradation of anthocyanins [6]. Consequently, 200, 300, and 400 W ultrasound power were used in the subsequent experiments.

The extraction temperature is one of the vital parameters that influence the yield of anthocyanins from blueberry wine residues. Figure 2c displayed the effect of extraction temperature on the anthocyanins yield. The yield of anthocyanins substantially increased with the increasing extraction temperature up to a maximum of (8.82 ± 0.20 mg/g) (*p* < 0.05). At 50 °C, a marked decrease in anthocyanins yield was observed (*p* < 0.05). Initially, the reason might be due to the elevated temperature, which could improve the solubility of anthocyanins in the extraction solvent and accelerate the mass transfer of intracellular anthocyanins [15]. However, a high temperature may cause the degradation of anthocyanins because of its thermal susceptibility [6]. Similar experimental results were obtained by other authors in the case of anthocyanins from blackcurrants and blueberries by using different temperatures [16,17]. Therefore, the extraction temperatures of 45, 50, and 55 °C were used in follow-up experiments.

Many studies showed that the extraction time could affect the yield of anthocyanins. Experiments were conducted at various extraction times to investigate its influence on the extraction process. Figure 2d showed that the initial yield of anthocyanins remarkably increased with increasing of the extraction time up to 30 min (*p* < 0.05), because in the early stage of extraction the intracellular anthocyanins had a low diffusion resistance due to the ultrasound-damaged cell structure, which was conducive to anthocyanins extraction [6]. Nevertheless, a prominent decrease in the yield of anthocyanins from blueberry wine residues was observed when the extraction time was over 30 min (*p* < 0.05). This phenomenon could be because the excessive extraction time led to the oxidative degradation of anthocyanins [11]. Hence, the extraction times used for the subsequent experiments were 20, 30, and 40 min.

### 2.3. Modeling of the Extraction Process

The experimental design based on RSM coupled with Box–Behnken design (BBD) and data of 29 runs are shown in Table 1, and the variance analyses of experimental results are shown in Table 2. Experimental results showed that the linear coefficient of water content (*X*_1_) was significant (*p* < 0.05), whereas the ultrasonic power (*X*_2_), extraction temperature (*X*_3_), and extraction time (*X*_4_) were extremely considerable (*p* < 0.01). In addition, the water content (*X*_1_) and the ultrasonic power (*X*_2_) involved in the experiment had extremely remarkable quadratic effect on anthocyanins yield (*p* < 0.01). The interaction terms of *X*_1_*X*_2_ and *X*_2_*X*_3_ had highly prominent effects on the anthocyanins yield (*p* < 0.01). The other interaction parameters had no notable effect on the anthocyanins yield (*p* > 0.05). Non-significant factors were initially removed, and the multiple regression analysis was used on the experimental data. The predicted anthocyanins yield was calculated using Equation (1).

(1) $$\begin{array}{l} Y=8.51+0.26X_{1}+0.36X_{2}+0.35X_{3}+0.55X_{4}-0.46X_{1}X_{2}\\ +0.56X_{2}X_{3}-0.36X_{1}^{2}-0.63X_{2}^{2} \end{array}$$

The *p*-value and the *F*-value were employed to evaluate the importance of each parameter. Low *p*-value and high *F*-value suggested that the related experimental factors were highly remarkable. Liu et al. (2013) have confirmed that the model was conspicuous and could promote the experimental factors when the *p*-value was less than 0.01 [18]. The relationship between the description of the above regression model and the response face value was extremely marked (*p* < 0.0001). However, the lack of fit was not prominent (*p =* 0.2420 > 0.005). The total (*R*^2^) and the adjusted (*R*^2^_adj_) determination coefficients in the regression model were 0.9099 and 0.8198, respectively, which implied a high relevance between the experimental and the predicted values (Table 2). The coefficient of variation (CV) and the percentage of absolute error of deviation (AED) were 0.5407% and 3.18%, respectively, which indicated that the regression model was reasonable and accurate. It is widely accepted that the model should be considered as reasonably reproducible if the CV value and AED value are lower than 10%. Consequently, the regression model had adequately represented the real relationship between experimental factors and the anthocyanins yield and could be used to obtain the optimal extraction parameters for the UADESE anthocyanins from blueberry wine residues.

**Figure 3:**
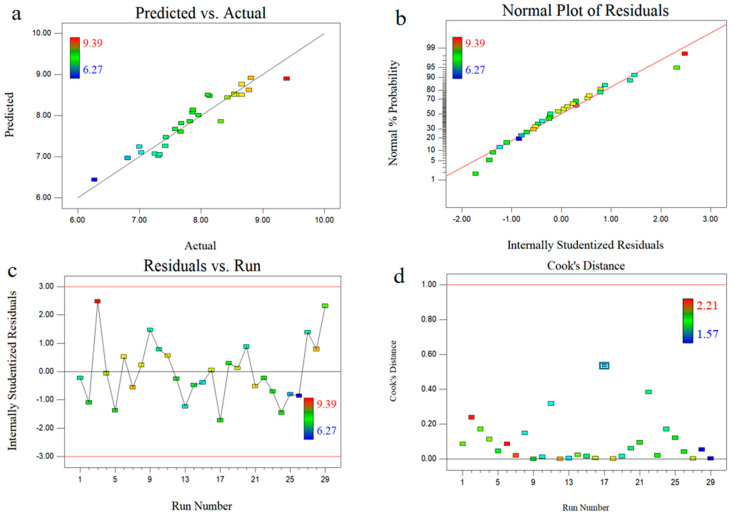
Diagnostic plots for model adequacy. Predicted versus actual (**a**), normal % probability (**b**), internal residuals (**c**), and Cook’s distance (**d**).

Figure 3a shows that the predicted values were consistent with the actual values, indicating that the predicted values could reasonably explain the experimental values. As shown in Figure 3b, these values also followed the normal distribution and had no deviation from the variance. In addition, the plots of the residuals and all data points were within ±3 (Figure 3c). Figure 3d shows the Cook’s distances of the simplified polynomial model. Cook’s distances were less than the limit of 1.0 so that there were no outliers in the given dataset.

### 2.4. Interaction of Process Variables

**Figure 4:**
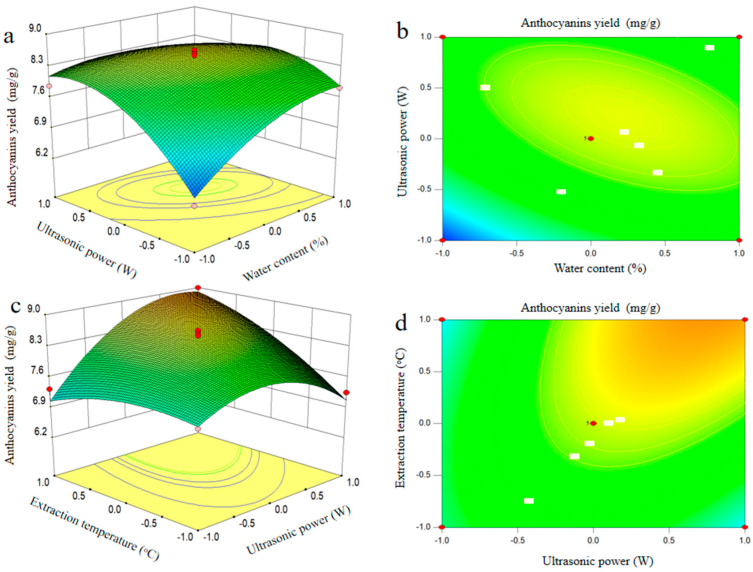
Three-dimensional response surface plots and corresponding contour plots. Influence of water content and ultrasonic power (**a**,**b**), ultrasonic power and extraction temperature (**c**,**d**) on anthocyanins yield from blueberry wine residues.

The RSM chart shows the interaction between different experimental factors in the extraction process and tests the influence of the interaction between the other two factors on the anthocyanins yield when the other factors are set at the zero level. The three-dimensional response surface figure and two-dimensional contour plots were drawn in accordance with the regression model. The interaction of experimental factors is described in Figure 4. The three-dimensional response surface could directly reflect the effect of the interaction of various experimental factors on the yield of anthocyanins in blueberry wine residues. The steep three-dimensional response surface showed that this experimental factor had a great influence on the yield of anthocyanins, and the interaction of the two experimental factors was highly marked. In the two-dimensional contour, the oval and the circular contours revealed that the interaction between the two factors was significant and not significant, respectively. Figure 4a shows the anthocyanins yield as a function of the water content (*X*_1_) and ultrasonic power (*X*_2_) when the extraction temperature (*X*_3_) and extraction time (*X*_4_) were fixed at the zero level. A marked interaction (*p* < 0.001) was observed between the water content (*X*_1_) and ultrasonic power (*X*_2_). The anthocyanins yield initially increased with increasing of water content (*X*_1_) and ultrasonic power (*X*_2_), and then decreased. The interaction caused by both water content (*X*_1_) and ultrasonic power (*X*_2_) in this study was in good agreement with the previous study [19]. Figure 4c depicted that the anthocyanins yield was influenced by both ultrasonic power (*X*_2_) and extraction temperature (*X*_3_). Moreover, Table 2 indicated a notable interaction between these variables at *p* < 0.01. The yield of anthocyanins also increased and reached the maximum when ultrasonic power (*X*_2_) and extraction temperature (*X*_3_) increased. Any further increase in these two variables showed the negative effect on anthocyanins yield. Bosiljkov et al. (2017) have observed a similar trend when they have investigated the effect of UADESE conditions on the yield of anthocyanins from wine lees [20].

### 2.5. Optimal Values from GA and Confirmation

**Figure 5:**
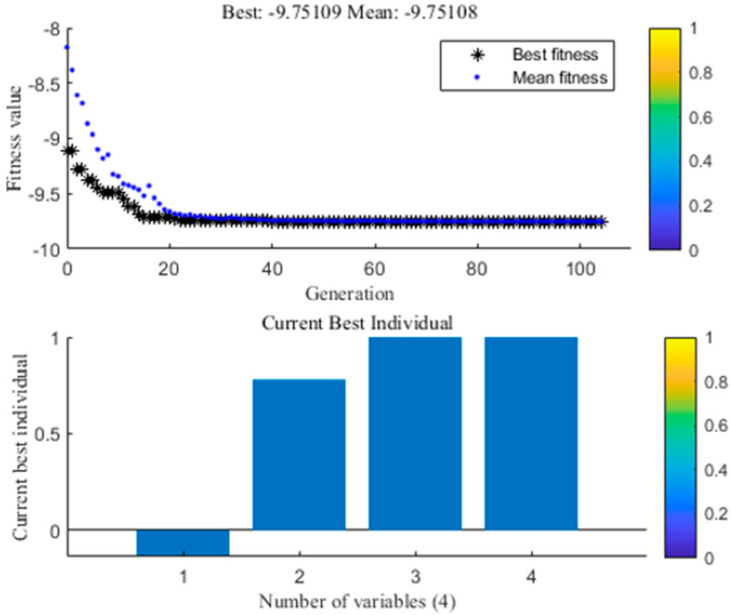
Best fitness and best individual graph.

GA is employed to achieve a global solution for the multiple regression model based on RSM. The GA toolbox of the MATLAB version R2018b was employed in optimization studies. The running results of the GA M file are shown in Figure 5. The optimum extraction parameters to achieve the highest yield of anthocyanins (9.75 mg/g) from blueberry wine residues by using the UADESE were obtained at water content of 28.63%, ultrasonic power of 378 W, extraction temperature of 55 °C, and extraction time of 40 min. Figure 5 described the best fitness value and level for each factor. The negative sign on the optimal fitness graph was due to the negative sign in the multiple regression model before running the GA. The best individual plot indicated the extraction time as the best individual. A confirmatory experiment was performed to verify the reliability of the GA. According to the actual situation, the above process parameters were modified as follows: water content of 29%, ultrasonic power of 380 W, extraction temperature of 55 °C and extraction time of 40 min. Under this condition, the experimental value of anthocyanins yield was (9.32 ± 0.08 mg/g). Results indicated that the experimental and the predicted values were in accordance with a 95% confidence interval. Thus, GA coupled with RSM was successfully used to optimize the extraction conditions for the UADESE anthocyanins from blueberry wine residues.

### 2.6. Identification of Anthocyanins from Blueberry Wine Residues

The chromatographic profiles of anthocyanins in the CE and the component Ⅰ at 525 nm obtained using HPLC-DAD are shown in Figure S1. In the HPLC-DAD analysis, five peaks were observed in CE (Figure S1a). However, component Ⅰ had a single peak (Figure S1b). Results indicated that the CE was a mixture with various anthocyanins components, and that the component Ⅰ was an individual anthocyanin. According to Equation (10), the purity of component Ⅰ was 92.81%. In accordance with the retention time, component Ⅰ corresponded to peak 2 in Figure S1a. Subsequently, HPLC-ESI-MS/MS was utilized to further analyze the anthocyanins components in CE. Identification process was performed by comparing the masses of molecular and fragment ions with the results reported in literature. The data obtained from the analysis of molecular ion, fragment ion, and retention time of anthocyanins peaks by HPLC-ESI-MS/MS are shown in Table 3 and the mass spectra of the anthocyanins compositions in CE are described in Figure S2.

The MS analysis of peak 1 (*t*_R_ = 12.60) showed an molecular ion at *m/z* 465.1 and a major fragmentation in MS^2^ at *m/z* 303.1, and the fragment (M^+^–162) corresponded to the loss of a glucose or galactose moiety (Table 3). The MS^2^ fragmentation of the ion at 303 indicated a delphinidin aglycone moiety. These results agreed with those of other studies [21,22]. Hence, peak 1 was tentatively identified as delphinidin-3-glucoside.

Subsequently, peak 2 (*t*_R_ = 19.36) showed an molecular ion at *m/z* 595.4. The two MS^2^ fragmentations detected in mass spectrometry were *m/z* 449.1 and 287.1, respectively (Table 3). The MS^2^ fragmentation of ion at *m/z* 287.0 corresponded to the cyanidin aglycone moiety. The fragment ([M–146–162]^+^) corresponded to the loss of a glycoside on *m/z* 449.0. The mass spectra obtained were similar to those obtained by other authors in the case of cyanidin-3-rutinoside from litchi pericarp and tartary buckwheat [23,24]. Thus, peak 2 (component Ⅰ) was tentatively identified as cyanidin-3-rutinoside. 

Peak 3 (*t*_R_ = 28.12) appeared to the molecular ion at *m/z* 479.3 with fragmentation patterns (*m/z* 317.1, [M–162]^+^), which corresponded to the loss of a glucose or galactose moiety (Table 3). The MS^2^ fragmentation with an ion at *m/z* 317 corresponded to the petunidin aglycone moiety. The nature of the hexose was determined by comparing the retention time with the data reported in previous studies [25,26]. Therefore, peak 3 was tentatively identified as petunidin-3-glucoside.

The ESI/MS profile of peak 4 (*t*_R_ = 34.44) presented the molecular ion at *m/z* 625.2. Two MS^2^ fragmentations detected in mass spectrometry were *m/z* 463.1 and *m/z* 301.3, respectively (Table 3). The fragment ion peak [M−162−162]^+^ at *m/z* 301, which was obtained by losing two hexoses (162), was referred to as peonidin. Therefore, peak 4 was tentatively identified as peonidin-3,5-dihexoside. Peak 5 (*t*_R_ = 37.97) with molecular ion at *m/z* 493.1 was tentatively identified as malvidin-3-glucoside. The fragment ion peak [M−162]^+^ at *m/z* 331.0 corresponded to the loss of the glucoside from malvidin-3-glucoside. This anthocyanin was identified in grape skins [27], *Rhodomyrtus tomentosa* [28] and *Vitis vinifera* red grape [29].

### 2.7. Effect of CE and Component Ⅰ on Cell Viability

**Figure 6:**
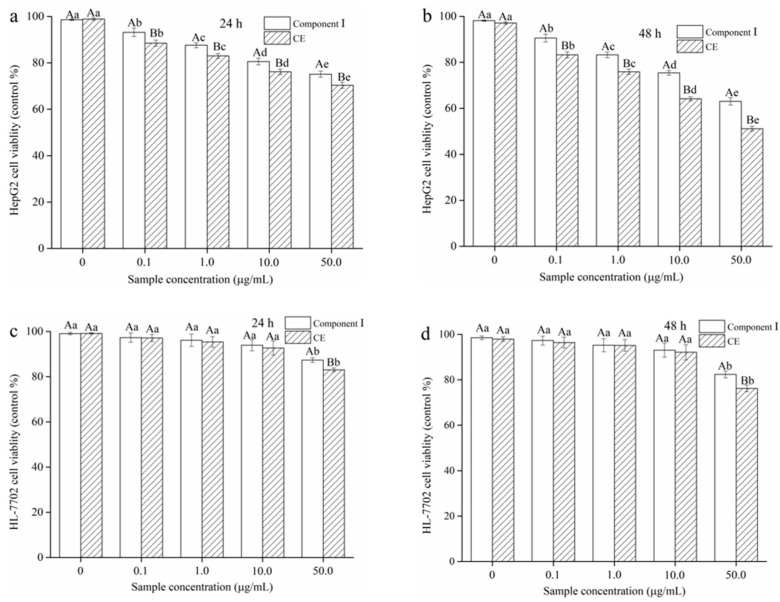
Effect of different concentrations of crude extract (CE) and component Ⅰ on the cell viability of HepG2 cells at (**a**) 24 h and (**b**) 48 h and HL-7702 cells at (**c**) 24 h, (**d**) 48 h. Note: Different lowercase letters indicate significant differences among different sample concentrations (*p* < 0.05), and different capital letters indicate that there are significant differences among different samples of the same concentration (*p* < 0.05).

First, the effects of the CE and component Ⅰ on cell viability were determined using the MTT assay. Experimental results are shown in Figure 6. The viability of HepG2 cells dramatically diminished with increasing of CE and component Ⅰ concentrations from 0.1 to 50.0 µg/mL at 24 and 48 h (*p* < 0.05). However, the viability of HepG2 cells treated with CE at different concentrations was significantly lower than that of the cells treated with component Ⅰ (*p* < 0.05). Moreover, the viability of the HepG2 cells treated with CE at 48 h was lower than that at 24 h. Results indicated that the anti-tumor effect of CE on HepG2 cells was better than that of component Ⅰ, and this effect was observed in a time-dependent manner. Based on the above results, the 48 h exposure of CE was chosen for later studies. In addition, the normal human hepatocytes (HL-7702 cells) were treated by using different concentrations of CE and component Ⅰ at 24 h and 48 h. Results are described in Figure 6c,d. The CE and the component Ⅰ had no notable effect on the viability of HL-7702 cells at concentrations ranging from 0.1 to 10.0 µg/mL at 24 and 48 h (*p* > 0.05). Results implied that CE and component Ⅰ had no toxic effect on HL-7702 cells at a range of 0.1–10.0 µg/mL. Subsequently, the cytotoxicity of the CE and the component Ⅰ to HL-7702 cells increased significantly with increasing concentrations at 24 and 48 h (*p* < 0.05). Consequently, 0.1–10.0 µg/mL of CE were used in subsequent experiments.

### 2.8. Effect of CE on ROS Production in HepG2 Cells

**Figure 7:**
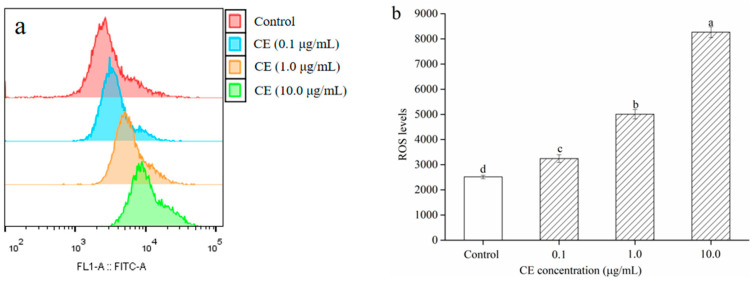
Effect of CE on the intracellular ROS levels in HepG2 cells for 48 h. ROS was determined by fluorescence microscope (**a**) and flow cytometry (**b**) detection. Note: Different lowercase letters indicate significant differences between groups, *p* < 0.05.

The production and the clearance of ROS in normal cells were in a dynamic equilibrium. The appropriate amount of ROS can promote macrophages to play their phagocytotic and enzymatic immunophysiological functions. However, the generation of excessive ROS can inhibit tumor cell activity and cause apoptosis. Thus, the fluorescent probe DCFH-DA was utilized to monitor the intracellular ROS levels and clarify whether the growth inhibition of CE on HepG2 cells was related to intracellular ROS levels. As illustrated in Figure 7, the pre-treatment of HepG2 cells with CE (0.1–10.0 μg/mL) for 48 h prominently increased the levels of intracellular ROS generation in a dose-dependent manner when compared with the vehicle treated group (*p* < 0.05). Results indicated that CE could inhibit the growth of HepG2 cells by improving the intracellular ROS levels. Similar experimental results were found in the case of purple rice and bilberry anthocyanins extracts [30,31].

### 2.9. Effect of CE on the Apoptosis of HepG2 Cells

**Figure 8:**
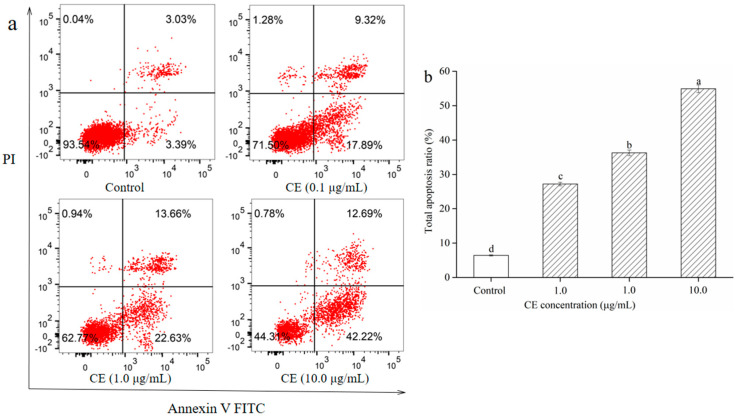
Effect of CE on apoptosis of HepG2 cells for 48 h. Apoptosis flow chart of HepG2 cells (**a**) and analysis of statistical results of flow cytometry detection (**b**). Note: Different lowercase letters indicate significant differences between groups, *p* < 0.05.

The Annexin V/PI double staining and the flow cytometry analysis were carried out to elucidate the effect of CE on the apoptosis of HepG2 cells. As shown in Figure 8, the percentage of apoptotic HepG2 cells in the control group was 6.42% ± 0.18% of the total cells. After exposure to 0.1, 1.0, and 10.0 μg/mL CE for 48 h, the percentages of apoptosis were 27.21% ± 0.53%, 36.29% ± 0.81%, and 54.91% ± 1.12%, respectively. Compared with that of the control group, the apoptosis rate of cells treated with 0.1, 1.0, and 10.0 μg/mL CE increased by 20.97%, 30.05%, and 48.67%, respectively. Results suggested that the CE had antitumor activity by promoting cell apoptosis. These experimental results were in good agreement with Long et al.’s (2018) results [32]. The mechanism might be that CE could accelerate the apoptosis of cancer cells by up-regulating the expression levels of apoptosis-related proteins and down-regulating the apoptotic inhibitory factors [33].

### 2.10. Effect of CE on the Cell Cycle of HepG2 Cells

**Figure 9:**
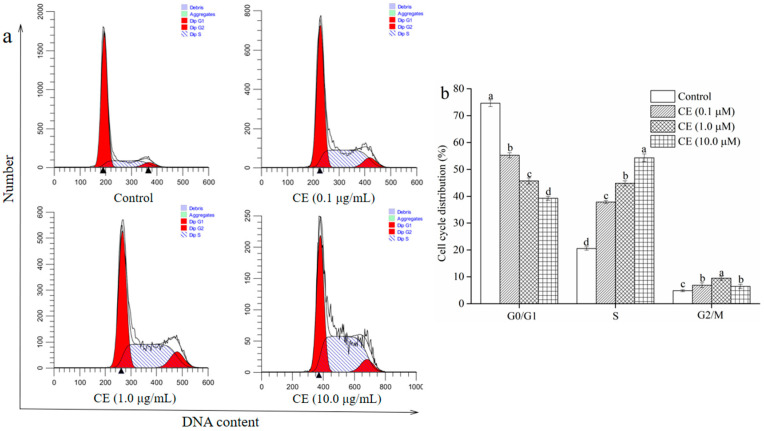
Effect of CE on cell cycle in HepG2 cells for 48 h. Cell cycle flow chart of HepG2 cells (**a**) and analysis of statistical results of flow cytometry detection (**b**). Note: Different lowercase letters indicate significant differences between groups, *p* < 0.05.

The effect of different concentrations of CE on the cell cycle distribution was analyzed using the commercial cell cycle detection kit to clarify whether the growth inhibition of CE on HepG2 cells was related to cell cycle arrest. As shown in Figure 9b, the percentage of HepG2 cells in the G0/G1 phases was dramatically reduced when cells were exposed to CE (0.1–10.0 μg/mL) for 48 h (*p* < 0.05). The CE treatment (0.1–10.0 μg/mL) notably enhanced the percentage of cells in the S phases in a dose-dependent manner compared with the vehicle-control treatment (*p* < 0.05). Compared with the vehicle treated group, 0.1–10.0 μg/mL CE treatment significantly increased the percentage of cells in the G2/M phases (*p* < 0.05). Nevertheless, no remarkable difference in the percentage of cells in the G2/M phases was observed between the 0.1 μg/mL and the 10.0 μg/mL CE treated groups (*p* > 0.05). These results revealed that CE could arrest the HepG2 cells in the S phases.

## 3. Materials and Methods

### 3.1. Experimental Materials

Blueberry wine residues were collected from Heilongjiang blueberry manor Biotechnology Co.; Ltd. (Yichun, Heilongjiang Province, China). The squeezed blueberry wine residues weighing 50 kg were dried in the FD-1A-50 freezing-vacuum dryer (Yancheng, Jiangsu Province, China) at −18 ℃ until the moisture content was less than 5%, and then the dehydrated blueberry wine residues were stored at −18 ℃ for 12 h before crushed. The frozen samples were thawed and milled into powdered particles smaller than 0.45 mm using an electric mill (HK-600, Hongke Food Machinery Co.; Ltd., Yongkang, Zhejiang Province, China), sealed in a brown reagent bottle and stored at 4 ℃ for the follow-up experiments.

### 3.2. Reagents and Solvents

Cyanidin-3-O-glucoside (C3G) was offered from Chengdu Gelip Biotechnology Co.; Ltd. (Chendu, Sichuan Province, China). Choline chloride, glycerol, 1,3-butanediol, 1,4-butanediol, glycol, acetonitrile, and sodium acetate buffer of analytical purity were purchased from Shanghai McLean Biochemical Technology Co.; Ltd. (Shanghai, China). AB-8 macroporous resin and Sephadex LH-20 were offered from Tianjin Tianxin Fine Chemical Factory (Tianjin, China). Human hepatoma cells (HepG2 cells) and Human normal hepatic cell (HL-7702 cells) were afforded from Concorde Cell Repository (Beijing, China). The fetal bovine serum (FBS) was from GIBCO-BRL (Grand Island, NY, USA). MTT was obtained from Beijing Baileibo Technology Co.; Ltd. (Beijing, China). RMPI-1640 medium, penicillin, and streptomycin were provided from Beijing Shengmu Biotechnology Co.; Ltd. (Beijing, China). Trypsin was obtained from Beijing Fubo Biotechnology Co.; Ltd. (Beijing, China). Annexin V-FITC/PI cell apoptosis detection and ROS kits were from Shanghai Yisheng Biotechnology Co.; Ltd. (Shanghai, China). Formic acid, ethanol, and potassium chloride buffer were purchased from Shanghai Jinjinle Industrial Co.; Ltd. (Shanghai, China).

### 3.3. Preparation of DESs

DESs were prepared following the previous research with some modifications [11]. DESs consisted of two components with the specific ratios of HBA (choline chloride) to HBDs (glucose, 1,3-butanediol, 1,4-butanediol, glycol, and glycerol). In accordance with the proportion in Table 4, HBA and HBDs were well mixed, and the mixtures were stirred fully in a flask placed in a water bath maintained at 80 °C until a colorless homogeneous liquid was obtained.

### 3.4. UADESE Procedures

Anthocyanins were extracted using UADESE in accordance with a previously described method with some modifications [11]. The lyophilized blueberry wine residues (1000 g) were mixed with the prepared DESs at a solid-to-liquid ratio of 1:20 g/mL and carried out using the RY-TQ-C ultrasound equipment (Beijing Yaou Depeng Technology Co.; Ltd., Beijing, China). The ultrasonic power, extraction time, and temperature were set through the control panel of ultrasonic equipment. Thereafter, the extracted samples were centrifuged at 3000× *g* and 25 °C for 15 min by using the KH20R-Ⅱ type centrifuge (Hunan Kaida Scientific Instrument Co.; Ltd., Changsha, Hunan Province, China) to obtain the anthocyanins extract. The extract was concentrated using the DZFY-2L type vacuum rotary evaporator (Shanghai Xingke Instrument Co.; Ltd., Shanghai, China) at 40 °C and freeze-dried using the FD-1A-50 dryer for 48 h. After drying, the crude anthocyanin powder was sealed in a brown reagent bottle and stored at −18 °C.

### 3.5. Determination of Anthocyanins Yield

The yield of anthocyanins in blueberry wine residues was measured by a pH differential method using a UV-3802S visible spectrophotometer (Hangzhou, Zhejiang Province, China). Briefly, 3 mL of anthocyanins extracts were diluted with 5 mL of two different buffers (0.025 M KCl-HCl buffer at pH 1.0 and 0.4 M CH_3_COONa-HCl buffer at pH 4.5). Each of these mixtures was stored away from light for 30 min at 25 °C. The absorbance of each sample was determined at 510 and 700 nm, respectively. The anthocyanins yield was calculated by Equation (2) [34].

(2) $$c=\frac{\left[\left(A_{510}-A_{700}\right)pH_{1.0}-\left(A_{510}-A_{700}\right)pH_{4.5}\right]\times M_{\omega }\times DF\times V\times 1000}{\varepsilon \times L\times m}$$

where *M_ω_* is the molecular weight of C3G, 449.2 g/mol; *DF* is the dilution factor; *ε* is the molar extinction coefficient of C3G, 26,000 L/mol·cm; *L* is the path length, cm; *V* is the total volume of extraction solvent, mL; *m* is the weight of blueberry wine residues powder, g.

### 3.6. Experimental Design

#### 3.6.1. Screening of Variables

Choosing a suitable range of experimental factors contributes to the improvement of the yield of anthocyanins in blueberry wine residues. In this study, 300 g blueberry wine residue powder was used as the experimental material. Factors and their ranges were selected on the basis of our pre-experiment results. The water content in DESs of 10%–50%, ultrasonic power of 100–500 W, extraction temperature of 40–60 °C, and extraction times of 10–50 min were initially applied to single-factor experiments. Each single-factor experiment was repeated thrice, and experimental results were presented as mean ± standard deviation (SD).

#### 3.6.2. Box–Behnken Design (BBD)

According to the single-factor experimental results, the Box–Behnken design (BBD) of 4-factor-3-level was conducted to investigate the influence of different variables and their interactions on the yield of anthocyanins from blueberry wine residues. The following four independent factors were considered: water content in DESs (*X*_1_), ultrasonic power (*X*_2_), extraction temperature (*X*_3_), and extraction time (*X*_4_) are selected as independent variables, whereas the anthocyanins yield (*Y*) is regarded as the response. The level and code of these factors are shown in Table 5. All experiments were performed in triplicate. The experimental results were fitted into the full second-order polynomial model to obtain the regression coefficients, as presented in Equation (3) [35].

(3) $$Y=\beta_{0}+\sum_{j=1}^{k}\beta_{j}X_{j}+\sum_{j=1}^{k}\beta_{jj}X_{j}^{2}+\sum_{i}\sum_{<j=2}^{k}\beta_{ij}X_{i}X_{j}+e_{i}$$

where *Y* is the anthocyanins yield, mg/g; *X_i_* and *X_j_* are the coded variables (*i* and *j* range from 1 to *k*); *β*_0_, *β_j_*, *β_jj_* and *β_ij_* are regression coefficients of intercept coefficient, linear, quadratic, and the second-order terms, respectively; *k* is the number of independent parameters (*k* = 4) and *e_i_* is the error.

#### 3.6.3. Validation of Model

The regression coefficient (*R*^2^) and the percentage absolute error of deviation (AED) between the experimental (*Y*_exp_) and the calculated (*Y*_mod_) results were used to assess the effectiveness of the model in this paper. *R*^2^ and AED were calculated using Equations (3) and (4), respectively.

(4) $$\mathrm{AED}(\%)=\frac{100}{p}\sum_{i=1}^{p}\left|\frac{Y_{\exp}-Y_{\mathrm{mod}}}{Y_{\exp}}\right|$$

### 3.7. GA

GA is a new generation of heuristic algorithm to simulate the genetic evolution of natural organisms. According to the set fitness function, mating, mutation, and fitness evaluation, it has a strong screening ability to avoid the limitations of the traditional Lagrangian algorithm and achieve the goal of global optimal solution. 

#### 3.7.1. Establishment of the Mathematical Model

The optimization method is used to analyze the constraints and decision variables in UADESE anthocyanins from blueberry wine residues. The objective function and mathematical model are developed according to the process index. The establishment of a mathematical model is divided into the following three steps.

(1) Determination of decision variables

According to the single-factor experimental results, four factors were selected as decision variables: Water content in DESs (*X*_1_), ultrasonic power (*X*_2_), extraction temperature (*X*_3_), and extraction time (*X*_4_). The process could be represented by Equation (5).

(5) $$X=X\left(X_{1},X_{2},X_{3},X_{4}\right)$$

The optimal solution can be found in many different schemes by solving the *X* of the relevant objective function under the constraint conditions. The optimal process parameters of anthocyanins from blueberry wine residues by UADESE can be ultimately determined.

(2) Determination of objective function

Deviation method has the advantage of avoiding the influence of unit dimension on optimization model. The process could be described by Equation (6)

(6) $$K^{i}=K_{d}^{i}/K_{m}^{i}$$

where *K^i^_d_* is the design value of the *i*-th parameter; *K^i^_m_* is the target value of the *i*-th parameter; *K^i^* is value of *i*-th parameter processing.

When *K^i^* is equal to 1, the function exactly satisfies the design aim of the objective parameter *i*. Euclidean distance is employed to express the deviation of each parameter from the target value, which is calculated by Equation (7).

(7) $$E^{i}={\left(1-k^{i}\right)}^{2}$$

where *k^i^* is the deviation of parameter *i* from the target value. Each design parameter has a big optimization objective. The following objective function is confirmed to achieve overall optimization.

(8) $$\mathrm{Maximize}\;f\left(x\right)=\sum w^{i}e^{i}$$

where *w_i_* is the target weight of the *i*-th parameter, $\sum w^{i}=1$. When *w_i_* is larger, parameter *i* is more important, and the goal of parameter *i* is satisfied first in design.

(3) Determination of constraint function

Constraints of GA optimization: Select the upper and lower limits of each factor level, and the constraints of the highest anthocyanins yield are as follows:

(9) $$\{\begin{array}{c} 20\%\leq X_{1}\leq 40\%\\ 200\;W\leq X_{2}\leq 400\;W\\ 45{\;}^{o}C\leq X_{3}\leq 55{\;}^{o}C\\ 20\;\min\leq X_{4}\leq 40\;\min \end{array}$$

#### 3.7.2. Mathematical Model Solution

The optimization mathematical model of UADESE anthocyanins from blueberry wine residues is a mixed programming problem with inequality constraints. According to the required optimization objective function and related constraints, the GA toolbox of MATLAB version R2018b was employed for optimization studies. The objective function can be detected by Equation (8).

### 3.8. Purification of Anthocyanins in Blueberry Wine Residues

The separation and the purification of anthocyanins were described in a previous report [36]. CE (200 mL) was slowly added to a pre-treatment AB-8 resin column (2.6 cm × 60 cm) at a flow rate of 3 BV/h. The column that absorbed anthocyanins was eluted with ultrapure water at 4 BV/h to remove the impurities, including sugars, proteins, and polar compounds, followed by elution with 70% ethanol (ethanol:water, *v/v*) of pH 3 at 2 BV/h. The eluent was collected, and ethanol was recovered using the rotary evaporator at 40 °C to obtain an anthocyanin-rich solution. The anthocyanin-rich solution was further separated using Sephadex LH-20 medium pressure column chromatography (2.9 × 25 cm) and washed with 70% ethanol (ethanol:water, *v/v*) at 1.5 BV/h when anthocyanins were completely absorbed in the Sephadex LH-20. The eluent was collected 1 tube for pre 3 mL and freeze-dried via the FD-1A-50 dryer. Finally, the purified anthocyanin component (component Ⅰ) was obtained.

### 3.9. HPLC-Diode Array Detector (DAD) and HPLC-ESI-MS/MS for Anthocyanins

The HPLC-DAD and HPLC-MS methods for the analysis of anthocyanins components in CE and component Ⅰ were consistent with our previous study [11]. Before the analysis, the CE and the component Ⅰ were dissolved in 5 mL HCl-methanol solution (0.1% (*v/v*)) and filtered through a 0.45 μm filter membrane. The 1100 series liquid chromatography system equipped with a DAD and Zorbax Eclipse XDB-C_18_ column (4.6 × 150 mm, 5 μm, Agilent) was used to determine the anthocyanins contents. Two solvents, including 5% (*v/v*) formic acid (mobile phase A) and 1% (*v/v*) formic acid acetonitrile (mobile phase B), were used in the mobile phase. The gradient elution was as follows: 5%–20% B (0–5 min), 20%–25% B (5–15 min), 25%–30% B (15–20 min), 30%–33% B (20–35 min), and 33%–5% B (35–40 min). The mobile phase was pumped by the system at a rate of 0.8 mL/min. The injection amount of the sample was set to 20 µL. Moreover, the column temperature and the wavelength set 25 °C and 520 nm, respectively. The purity of the anthocyanins component can be calculated using Equation (10).

(10) $$w_{i}(\%)=\frac{f_{i}A_{i}}{\sum f_{i}A_{i}}$$

where *w_i_* and *A_i_* are purity and peak area of component *i*, respectively. *f_i_* is the correction factor.

The mass spectra were obtained at a range of 100–1000 *m*/*z* in the positive mode. The voltages of the capillary, sampling cone, and extraction cone were 2.0 kV, 40 V, and 2.0 V, respectively. The temperatures of source and desolvation were set to 115 and 350 °C, respectively. The time of scan and interscan were 13 min and 0.28 s, respectively. The gas flow of the cone and the desolvation were 50 and 900 L/h, respectively. The collision energy was set 20.0–45.0 eV. The Mass-Lynx TM V 4.1 software was used to collect experimental data.

### 3.10. Antitumor Activity

#### 3.10.1. Cell Culture and Drug Preparation

HepG2 and HL-7702 cells were cultured in the RMPI-1640 medium with 10% FBS and 1% penicillin-streptomycin and placed in an incubator maintained at 37 °C and 5% CO_2_, and the media were replenished every two days. Logarithmic cells were used in this study. The RMPI-1640 medium was employed to prepare the sample solutions of CE and component Ⅰ. Samples were filtered through a 0.22 µm membrane for sterilization before the experiment.

#### 3.10.2. Cell Viability

Cell viability was measured by using the MTT method in accordance with the previously described method [37]. The HepG2 and HL-7702 cells (5 × 10^3^ cells/mL) were grown in 96-well plates at 37 °C in a humidified 5% CO_2_ incubator for 24 h. Cells were treated with different concentrations of CE (0.1, 1.0, 10.0, and 50.0 μg/mL) and component Ⅰ for 24 h and 48 h. After the above treatment, the supernatant was removed, and each well was added with 20 µL MTT (5 mg/mL) and incubated for another 4 h. The culture medium was then replaced with 150 µL DMSO in each well to dissolve the formed blue formazan crystals. Subsequently, the absorption value of each well was determined at 490 nm by using the WD-2102b automatic enzyme labeling instrument (Linyi, Shandong Province, China). Cell viability was calculated using Equation (11). All experimental results were described as the mean ± SD of three experiments with six wells per treatment group.

(11) $$\mathrm{Cell}\;\mathrm{viability}/\%=\frac{\mathrm{Absorbance}\;\mathrm{of}\;\mathrm{sample}\;\mathrm{group}}{\mathrm{Absorbance}\;\mathrm{of}\;\mathrm{control}\;\mathrm{group}}\times 100\%$$

#### 3.10.3. Determination of Intracellular ROS

In accordance with the results of MTT, CE was selected for subsequent experiments. The effect of CE on ROS generation in HepG2 cells was monitored by the ROS kit in accordance with its instructions. In short, HepG2 cells (1 × 10^5^ cells/mL) were seeded into 6-well plates and incubated overnight. Cells were treated with different concentrations of CE (0, 0.1, 1.0, 10.0 μg/mL) for 48 h. Cells were then washed with PBS and subsequently incubated with 5 μM DCFH-DA in PBS at 37 °C for 30 min. HepG2 cells were washed with PBS, and collected by centrifugation, and re-suspended in PBS. Finally, the ZE5 multicolor flow cytometer (American Bio-rad Company, Hercules, CA, USA) was used to determine the fluorescence intensity.

#### 3.10.4. Apoptosis Assay

The apoptosis of HepG2 cells was determined using the Annexin V-FITC and propidium iodine (PI) dual staining, which was performed using the ZE5 multicolor flow cytometer [38]. The treated HepG2 cells were collected, washed thrice with ice-cold PBS, and slowly resuspended in Annexin V binding buffer, and the mixture was incubated with Annexin V-FITC and PI in the dark for 10 min, respectively. Ultimately, the samples were analyzed immediately using the ZE5 multicolor flow cytometer.

#### 3.10.5. Cell Cycle Analysis

The cell cycle analysis was conducted using a previously published method [39]. Briefly, HepG2 cells were grown into 6-well plates at a density of 1 × 10^5^ cells/mL and incubated overnight. The cells were exposed to different concentrations of CE (0, 0.1, 1.0, 10.0 μg/mL). After 48 h, HepG2 cells were harvested and fixed with cold 70% ethanol (ethanol:water, *v/v*) at −20 °C for 24 h. Subsequently, the treated HepG2 cells were washed with PBS and incubated with RNase (50 μg/mL) at 37 °C for 20 min. Finally, the cells were stained with 50 μg/mL PI at 4 °C for 15 min in the dark and subjected to the ZE5 multicolor flow cytometer. The experimental data were analyzed using the Modfit LT software (Version 5.0, Verity Software House, Los Angeles, USA).

### 3.11. Statistical Analysis

All experimental data were represented as mean ± SD. The one-way analysis of variance was used to analyze statistical differences between groups. *p* < 0.05 represented the experimental data with statistical significance. Design-Expert software (version 8.0.6) for BBD analyses was employed to design combinatorial experiments. All analyses were performed with SPSS Statistics 19 (version 13.0). Origin software (version 9.0) was used for drawing in this study.

## 4. Conclusions

This study was performed to investigate the UADESE anthocyanins from blueberry wine residues and optimize the extraction conditions by GA based on RSM. The optimum extraction parameters to achieve the highest yield of anthocyanins of (9.32 ± 0.08 mg/g) from blueberry wine residues via UADESE were obtained at water content of 29%, ultrasonic power of 380 W, extraction temperature of 55 °C, and extraction time of 40 min. The cyanidin-3-rutinoside with purity of 92.81% was obtained. The HepG2 antitumor activity of CE was better than that of component Ⅰ. In addition, CE could increase the intracellular ROS levels and the apoptosis, and arrest the HepG2 cells in the S phases. Finally, findings confirmed that the UADESE was an efficient, reliable, and environmentally friendly extraction method. Further studies will focus on more detailed identification and quantification of other phenolic compounds available in the extract and to investigate their antitumor mechanism.

## Figures and Tables

**Table 1:** Coded levels of independent variables in the Box–Behnken design and experimental observations.

No.	Independent Variables				Anthocyanins Yield (mg/g)
	Water Content (*X*_1_)/%	Ultrasonic Power (*X*_2_)/W	Extraction Temperature (*X*_3_)/°C	Extraction Time (*X*_4_)/min	
1	−1	−1	0	0	6.27
2	1	−1	0	0	7.82
3	−1	1	0	0	7.86
4	1	1	0	0	7.58
5	0	0	−1	−1	6.81
6	0	0	1	−1	7.87
7	0	0	−1	1	8.54
8	0	0	1	1	8.66
9	−1	0	0	−1	7.31
10	1	0	0	−1	8.32
11	−1	0	0	1	8.43
12	1	0	0	1	8.78
13	0	−1	−1	0	7.43
14	0	1	−1	0	7.25
15	0	−1	1	0	7.33
16	0	1	1	0	9.39
17	−1	0	−1	0	7.42
18	1	0	−1	0	7.68
19	−1	0	1	0	7.96
20	1	0	1	0	8.14
21	0	−1	0	−1	7.03
22	0	1	0	−1	7.00
23	0	−1	0	1	7.67
24	0	1	0	1	8.81
25	0	0	0	0	8.65
26	0	0	0	0	8.66
27	0	0	0	0	8.57
28	0	0	0	0	8.11
29	0	0	0	0	8.54

**Table 2:** Significance test report of the regression model coefficient of anthocyanins extracted from blueberry wine residues by ultrasonic-assisted deep eutectic solvents extraction (UADESE).

Source of Variance	*SQ*	*df*	*MS*	*F*-Value	*p*-Value
Model	13.21	14	0.94	10.10	<0.0001 **
*X*_1_	0.79	1	0.79	8.40	0.0117 *
*X*_2_	1.57	1	1.57	16.79	0.0011 **
*X*_3_	1.48	1	1.48	15.88	0.0014 **
*X*_4_	3.58	1	3.58	38.25	<0.0001 **
*X*_1_^2^	0.86	1	0.86	9.17	0.0090 **
*X*_2_^2^	2.57	1	2.57	27.52	0.0001 **
*X*_3_^2^	0.40	1	0.40	4.33	0.0564 ^ns^
*X*_4_^2^	0.16	1	0.16	1.69	0.2150 ^ns^
*X*_1_ *X*_2_	0.84	1	0.84	8.96	0.0097 **
*X*_1_ *X*_3_	1.600 × 10^−3^	1	1.600 × 10^−3^	0.017	0.8978 ^ns^
*X*_1_ *X*_4_	0.11	1	0.11	1.17	0.2986 ^ns^
*X*_2_ *X*_3_	1.25	1	1.25	13.42	0.0026 **
*X*_2_ *X*_4_	0.34	1	0.34	3.66	0.0764 ^ns^
*X*_3_ *X*_4_	0.22	1	0.22	2.36	0.1465 ^ns^
Residual	1.31	14	0.093		
Lack of fit	1.10	10	0.11	2.13	0.2420 ^ns^
Pure error	0.21	4	0.052		
Sum	14.52	28			
	*R*^2^ = 0.9099		*R*^2^_adj_ = 0.8198	CV = 0.5407%	

Note: * stands for significant difference (*p* < 0.05); ** represents highly significant difference (*p* < 0.01); ^ns^ represents no significant difference (*p* > 0.05); *SQ* is sum of squares; *df* is degree of freedom; *MS* is mean square deviation.

**Table 3:** Identification of anthocyanins in blueberry wine residues.

Peak	*t*_R_ (min)	(M)^+^ (*m/z*)	MS^2^ (*m/z*)	Lost Fragments (*m/z*)	Identity
1	12.60	465.1	303.1	162	delphinidin-3-glucoside
2	19.36	595.4	449.1, 287.1	146, 162	cyanidin-3-rutinoside
3	28.12	479.3	317.1	162	petunidin-3-glucoside
4	34.44	625.2	463.1, 301.3	162	peonidin-3,5-dihexoside
5	37.97	493.1	331.1	162	malvidin-3-glucoside

**Table 4:** Different types of DESs.

NO.	HBA	HBD	Mole Ratio	DESs:Water (*v/v*)
DESs-1	choline chloride	glycerol	1:2	80:20
DESs-2	choline chloride	glycerol	1:3	80:20
DESs-3	choline chloride	1,3-butanediol	1:2	80:20
DESs-4	choline chloride	1,3-butanediol	1:3	80:20
DESs-5	choline chloride	1,4-butanediol	1:2	80:20
DESs-6	choline chloride	1,4-butanediol	1:3	80:20
DESs-7	choline chloride	glycol	1:2	80:20
DESs-8	choline chloride	glycol	1:3	80:20
DESs-9	choline chloride	glucose	1:1	80:20
DESs-10	choline chloride	glucose	1:2	80:20

**Table 5:** Experimental design independent variables and their levels.

Levels	Independent Variables			
	Water Content (*X*_1_)/%	Ultrasonic Power (*X*_2_)/W	Extraction Temperature (*X*_3_)/°C	Extraction Time (*X*_4_)/min
−1	20	200	45	20
0	30	300	50	30
1	40	400	55	40

## Supplementary Materials

The following are available online, Figure S1: HPLC-DAD chromatogram of CE (a) and component Ⅰ (b), Figure S2: MS spectra of the anthocyanins components in CE.
