# Supplementary material for: Ultrasound-Assisted Deep Eutectic Solvent Extraction of Anthocyanins from Blueberry Wine Residues: Optimization, Identification, and HepG2 Antitumor Activity
Source: Molecules. 2020 Nov 20;25(22):5456. doi: 10.3390/molecules25225456 (PMC7699922; doi:10.3390/molecules25225456)
Supplement: Supplementary file 1 [file molecules-25-05456-s001.pdf]

Table of Contents

CONTENTS	page
**Fig. S1** HPLC-DAD chromatogram of CE (a) and component I (b).	2
**Fig. S2** MS spectra of the anthocyanins components in CE.	3

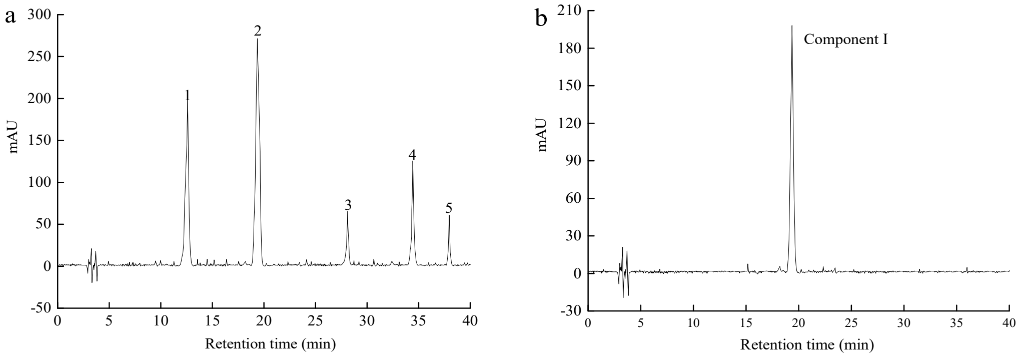
a
300
250
200
150
100
50
0
-50
mAU
1
2
3
4
5
0 5 10 15 20 25 30 35 40
Retention time (min)
b
210
180
150
120
90
60
30
0
-30
mAU
Component I
0 5 10 15 20 25 30 35 40
Retention time (min)

Fig. S1

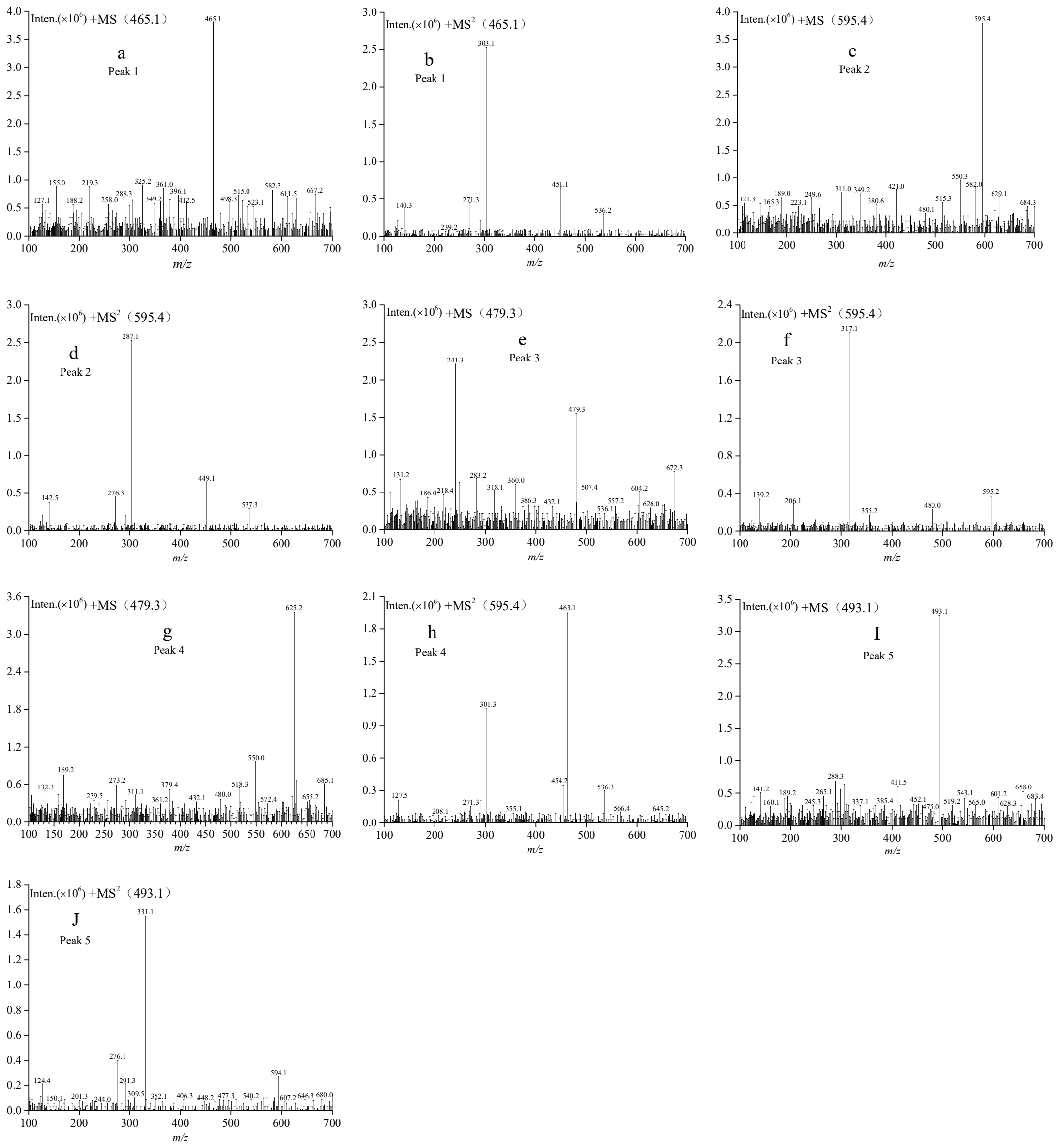
Inten.(×10⁶) +MS（465.1）
a
Peak 1
Inten.(×10⁶) +MS²（465.1）
b
Peak 1
Inten.(×10⁶) +MS（595.4）
c
Peak 2
Inten.(×10⁶) +MS²（595.4）
d
Peak 2
Inten.(×10⁶) +MS（479.3）
e
Peak 3
Inten.(×10⁶) +MS²（595.4）
f
Peak 3
Inten.(×10⁶) +MS（479.3）
g
Peak 4
Inten.(×10⁶) +MS²（595.4）
h
Peak 4
Inten.(×10⁶) +MS（493.1）
I
Peak 5
Inten.(×10⁶) +MS²（493.1）
J
Peak 5
m/z

Fig. S2
